# Heart rate variability: A biomarker of frailty in older adults?

**DOI:** 10.3389/fmed.2022.1008970

**Published:** 2022-10-14

**Authors:** Flávia Sousa Arantes, Vinicius Rosa Oliveira, Aime Karla Moraes Leão, João Pedro Ribeiro Afonso, Adriano Luis Fonseca, Daniela Rosana Pedro Fonseca, Diego Antonio C. Pina Gomes Mello, Ivan Peres Costa, Luiz Vicente Franco Oliveira, Renata Kelly da Palma

**Affiliations:** ^1^Human Movement and Rehabilitation, Post-Graduate Program Medical School, Evangelic University of Goiás-UniEVANGÉLICA, Anápolis, Brazil; ^2^Research Group on Methodology, Methods, Models and Outcomes of Health and Social Sciences (M_3_O), Faculty of Health Sciences and Welfare, Center for Health and Social Care Research (CESS), University of Vic-Central University of Catalonia (UVic-UCC), Vic, Spain; ^3^Department of Research, Innovation and Postgraduate, University of Rio Verde, Rio Verde, Brazil; ^4^Rehabilitation Sciences, University Nove de Julho (UNINOVE), São Paulo, Brazil; ^5^FacPhysio, São Paulo, Brazil; ^6^Department of Surgery, School of Veterinary Medicine and Animal Sciences, University of São Paulo, São Paulo, Brazil; ^7^Facultad de Ciencias de la Salud de Manresa, Universitat de Vic-Universitat Central de Catalunya (UVic-UCC), Manresa, Spain

**Keywords:** frailty, older adults, heart rate variability, autonomic control, wearable device (WD)

## Abstract

Frailty is a state of critical loss of physiological complexity resulting in greater vulnerability to stressors and has been characterized as a debility syndrome in the older adult. Changes in functional capacity and the cardiovascular system during aging are the most significant and relevant for this population, including the clinically healthy. In this sense, this review aims to investigate methods to monitor the performance of older adults, such as heart rate variability and verify how it can be related to frailty. It contributes to understanding that the changes in heart variability can be a marker for frailty in older adults.

## Introduction

The World Health Organization (WHO) established the period from 2021 to 2030 as the “Decade of healthy aging.” However, the COVID-19 pandemic demonstrated inequality in the aging process and the lack of public policies for this segment of the population, raising the importance of developing studies to alleviate the decline of intrinsic capacity associated with aging (1). The intrinsic capacity of older adults can be evaluated through performance measures, however, it remains a challenge to validate such measures in this population (2). Understanding the mechanism of vulnerability to stressors in frail older adults can become useful for the creation of preventive measures and improvement of quality of life and resistance to stressors (3). In this sense, this review aims to investigate methods to monitor the performance of older adults, such as heart rate variability and verify how it can be related to frailty.

## Frailty assessment

Frailty is a state of critical loss of physiological complexity resulting in greater vulnerability to stressors. This has been characterized as a debility syndrome in the elderly in which there is decreased strength, low physical activity, energy depletion and unintentional weight loss (4). In turn, frail older adults become more likely to develop health complications and a high risk of important adverse outcomes. They may also have accelerated functional decline, physical disability, low ability to recover and mortality (4).

**Figure 1:**
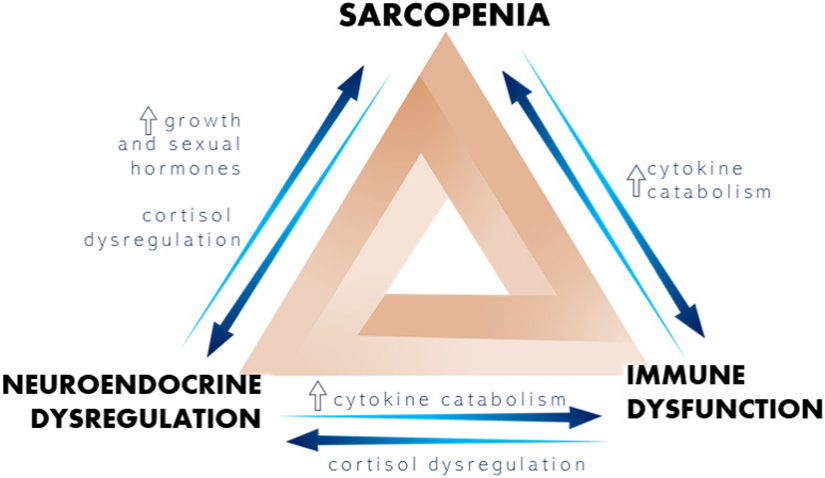
Representation of the most important physiological changes that trigger frailty.

As frailty develops in older adults, it often leads to a decline in general health, characterizing a dynamic state in which it can improve or worsen over time (5). For frailty assessment, different tools can be used but only a few of them divide the classification into pre-frailty, frailty and robust which allows us to apply preventive measures (Table 1), The most frequent tool is the frailty phenotype proposed by Fried et al. (7). The Fried Phenotype Criteria is determined by the presence of five measurable components, namely: (1) weakness measured by handgrip strength in the dominant hand; (2) slow gait; (3) unintentional weight loss greater than or equal to 4.5 kg or greater than 5% of body weight in the previous year; (4) report of exhaustion, assessed by self-report of fatigue, indicated by two questions on the Depression Scale of the Center for Epidemiological Studies, and (5) low level of physical activity (7). To be considered a frailty syndrome according to this index, three out of the five criteria must be present, in a way that those who present one or two criteria are considered pre-frail and those who do not obtain any are considered non-frail or robust (7). According to the theory of Fried et al. (7), frailty is based on a reduction in the activity of anabolic hormonal axes, the installation of sarcopenia and the presence of a chronic inflammatory state (Figure 1).

**Table 1:** Frailty assessment.

**Tools**	**Components**
FRAIL scale (6)	Fatigue, resistance, ambulation, illness, loss of weight
Frailty phenotype (7)	Weight loss, low physical activity, exhaustion, slowness, weakness
Study of Osteoporotic Fractures frailty criteria (8)	Weight loss, exhaustion, unable to rise from a chair five times
Multidimensional Prognostic Index (9) Clinical Frailty Scale (10)	Comorbidity, nutrition, cognition, polypharmacy, pressure score risk,living status, activities of daily living (ADL), instrumental activities of daily living (IAD) Basic activities of daily living (ADLs), instrumental ADLs, chronic medical conditions that require drugs, exercise, and appearing fitter compared with patients of similar age.

However, although the Fried phenotype is the most used tool to assess frailty, specially in patients with some heart disease (11), there are some challenges to be overcome. The Fried phenotype measuring can be complicated and relatively time-intensive and mainly focuses on physical impairments disregarding other domains such as cognitive dysfunction, which are common in older patients (12). In this sense, new methodology where combine physical and cognitive impairment evaluation should be consider for frailty assessment.

## Cardiovascular disease and frailty

Several studies have demonstrated the possible association between cardiovascular disease (CVD) and frailty (13–15). However, literature is limited to be sure if frailty can be a cardiovascular risk factor or vice versa. Many hypotheses are based on common risk factors to identify this association. Franceschi et al. (16), and Ferruci and Fabbri (17) suggested that the inflammatory process leads the cardiovascular diseases (CVD) and frailty. Furthermore, oxidative stress (18) and dysfunction in coagulation (13) are present in CVD and frailty. Even with all these associations, other mechanisms should be addressed, such as the activity of the autonomic system in frailty older adults. It is well documented that during the aging process there is a change in cardiovascular control, causing a decrease in vagal tone and an increase in sympathetic tone. These changes contribute to the occurrence of cardiovascular events, being one of the main causes of death in older adults (19).

## Heart rate variability methods of analysis

It is worth noting that parasympathetic activation is lower in older adults compared to younger individuals. There is evidence that even if the myocardium does not respond with an expected intensity concerning the increase in heart rate and contraction force, sympathetic modulation may be increased. In turn, these are some reasons why older adults have a higher cardiovascular risk, as changes in the autonomic balance can have serious consequences on health (20). Since de heart rate is modulated by sympathetic and parasympathetic system, Heart Rate Variability (HRV) can be considered a cardiac autonomic control marker (21). HRV is the quantitative measurement of minimal changes in heartbeats, which provides the regulation of the autonomic nervous system and reflects the system's ability to react to stressors. This index has gained prominence among the various cardiac health measurement indices (22, 23). In addition to the ability to coordinate between the sympathetic and parasympathetic nervous systems, HRV also acts as an indicator of other aspects directly linked to autonomic function, such as self-regulatory capacity, and psychological and physiological stress (23). HRV can be measured using an electrocardiogram (ECG) exam or a 24-h Holter monitor. Recent advances in technology, such as mobile apps, smartwatches, and other devices allow for less invasive and discreet assessments, without affecting the accuracy of the procedure (24).

The HRV calculation can be obtained through linear methods and two categories of measures have been used: time and frequency domain. The time domain, such as the R-R intervals (R-Ri), translate fluctuations in the duration of the cardiac cycle from statistical means. The statistical indices in the time domain include: SDNN (standard deviation of all R-Ri), SDANN standard deviation of the means of normal R-Ri every 5 min), rMSSD (square root of the mean square of the differences between adjacent normal R-Ri in a time interval) and pNN50 (percentage of R-Ri with duration difference >50 ms). The SDNN and SDANN represent the sympathetic and parasympathetic activities, but do not allow distinguishing when changes in HRV are due to increased sympathetic tone or withdrawal of vagal tone. The rMSSD and pNN50 indices represent parasympathetic activity (25).

Another linear method of analysis is the frequency domain, such analysis shows fundamental oscillatory components of the HRV, namely: High Frequency–HF (0.15 to 0.4 Hz corresponding to respiratory modulation, indicating vagal action under the heart); Low Frequency–LF (0.04–0.15 Hz, joint vagal and sympathetic action on the heart, with sympathetic predominance) and Very Low Frequency components–VLF, which seems to be related to the renin angiotensin aldosterone system, thermoregulation and peripheral vasomotor tone (25). For the selection of the appropriate index, the duration of the record and the quality of the data must be considered, carefully so as not to affect the results (23).

Time and frequency domain are measures which reflect the magnitude of heart rate fluctuation, and their decreases are associated with increased risk for cardiovascular disease. However, it has been shown that not all information carried by R-R intervals variability can be explained by linear method (26). Therefore, the nonlinear measures of HRV, can better capture the tiny but physiologically important changes in HRV and be associated with the development of cardiovascular disease as well (27). Nonlinear measures quantify properties of heart rate dynamics, caused by complex interplays between vagal and sympathetic regulations as response patterns and self-correlations (28) i.e., quantify the unpredictability of a time series. Some categories of nonlinear measures have been used: deceleration capacity (DC), estimating ability to decelerate heart rate on specific time scales, Poincaré plot, plotting every R–R interval against the prior interval, creating a scatter plot and fractal scaling exponents, assessing fractal organization of heart rate regulation based on chaos theory (28, 29). In Table 2 we summarize the linear and nonlinear measures of HRV.

**Table 2:** Methods of HRV measure.

**Method**	**Components**
**Linear**	
Time	• Standard deviation of all NN intervals total variability (SDNN) • Square root of the mean of the sum of the squares of differences between adjacent NN intervals (rMSSD) • Number of pairs of adjacent NN intervals differing by more than 50 ms in the entire recording (NN50 count) • NN50 count divided by the total number of all NN interval( pNN50)
Frequency	• Power in low frequency range ( ≤ 0.04 Hz) (LF) • Power in high frequency range (0.15–0.4 Hz) (HF) • LF power in normalized units LF/(Total Power–VLF) × 100 (LF nu) • HF power in normalized units HF/(Total Power–VLF) × 100 (HF nu) • Ratio LF /HF (LF/HF)
**Nolinear**	
Poincaré plot	• Area of the ellipse which represents total HRV (S) • Poincaré plot standard deviation perpendicular the line of identity (SD) • Poincaré plot standard deviation along the line of identity (SD2) • SD1/SD2 % Ratio of SD1-to-SD2
Deceleration capacity (DC)	• Detrended fluctuation analysis, which describes short-term (DFA α1) Fluctuations • Detrended fluctuation analysis, which describes long-term fluctuations (DFA α2) • Correlation dimension, which estimates the minimum number of variables required to construct a model of system dynamics (D2)
Fractal scaling exponents	• Approximate entropy, which measures the regularity and complexity of a time series (ApEn) • Sample entropy, which measures the regularity and complexity of a time series (SampEn)

## Evidence link between heart rate variability and frailty

Changes in functional capacity and the cardiovascular system during aging are the most significant and relevant for older adults (30). A systematic review conducted by Afilalo et al. (31) found that frailty increased 2 to 3-fold the risk of vascular disease. Additionally, other studies reported that increased frailty was correlated with increased cardiovascular risk and decreased survival (32–34).

Previous studies carried out with older women pointed out a correlation between HRV and frailty. Chaves et al. (3) used the non-linear measure of HRV [(ApEn) and Varadhan et al. (4) performed it through logarithmic transformation (SDNN, VLF, LF, and LF/HF)], demonstrated that decreases in HRV was associated with an increased risk of frailty. Katayama et al. (35) also found results similar to those mentioned previously, where differences in cardiac activity were found between frail and non-frail older women, which reinforced the theory of the influence of frailty on HRV, using linear (SD, RMSSD and LF/HF Ratio) and non-linear measurement (SampEn).

Another observational study showed that low HRV is related to physical frailty, indicating that this measure can add relevant information to assess physical functioning and identify individuals with a greater possibility of physical decline (23). Toosizadeh et al. (36), evaluated the HRV (RMSSD, HR mean and RR intervals) of older adults during gait, reporting the non-frail had a greater variety of HR concerning the frail and pre-frail. It is estimated that this difference is due to the lack of cardiovascular reserve and the impairment of the autonomic nervous system by the elderly in a situation of frailty or at the beginning of it. More recently, the same group carried out another study comparing the relationship between frailty and HRV variation during the performance of a functional task in older adults. This study showed that the recovery time of HR after the task was 47% lower in pre-frail/frail participants compared to non-frail, suggesting a strong association between the dynamics between HRV and frailty (37).

There is a link between low HRV and cognitive impairment, that acts as a biomarker due to autonomic dysfunction caused by dysregulation in cerebral perfusion. External factors such as cardiovascular risks are considered responsible for the association between HRV and frailty (38, 39). In addition, HRV may reflect an early manifestation of brain damage and future cardiovascular events. These events lead to cognitive decline through the cardiovascular regulatory processes in the brain and cognition regulatory processes located, especially, in the prefrontal cortex.

Reduced parasympathetic activity at rest has been related to worse performance on cognitive exercises, confirming the predictions of the “neurovisceral integration” model, which suggests that HRV can regulate the functional integrity of the central nervous system (40). Higher activities of prefrontal brain structures increase HRV, while underactivity reduces HRV. The predominantly vagal control of the heart allows flexible and rapid responses to environmental demands, promoting effective executive performance. Therefore, higher HRV is related to better cognitive performance, while low HRV has been associated with cognitive impairment and is considered an early biomarker of cognitive deterioration (40).

Therefore, we suggest that HRV measure can be used as a potential marker for frailty because it helps to understand the changes in cardiac autonomic modulation. Moreover, with the dates from HRV evaluation we can elaborate a strategy for prevent frailty and CVD. The idea of new methodologies with easy access to the population to assess HRV has been increasingly emerging.

## Methods to monitor the performance of the older adults

The changes in heart rate variability can be used as a marker for frailty and could be assessed using proper tools to monitor the heart rate variability in the older adult population. A systematic review by Parvaneh et al. (41) showed that frail compared to non-frail older adults present a reduction in the complexity of HR dynamics, reduced HRV, and reduced HR changes in response to daily activities (e.g., postural transitions from lying to standing). More recently, another systematic review revealed beneficial effects of monitoring HRV in healthy older adults during different exercise interventions (42). In this sense, wearable devices are non-invasive tools that present advantages such as low cost and high benefits.

The HR monitor RS800CX Polar Electro has been used successfully to measure the cardiac autonomic modulation in non-frail, pre-frail and frail elderly women (35). In addition, the use of Polar RS800 chest belt has also been reported in studies examining the effects of endurance training on various parameters of HRV in sedentary seniors (39, 43), and in a study of an exergaming-based dance training to improve HRV in healthy older adults (44). The Heart Rate Monitor Polar RS800 (45), Polar H7 Heart Rate Sensor (46) and Polar V800 Monitor (47) are one of the most well-established brands in HR monitoring, with Polar H7/H10 HR sensors having been validated both at rest and during exercise. The Polar V800 Monitor has been validated in detecting R-R intervals in the older adult population under mental stress or dual-task considerations (47).

Although the available evidence of wearable smart technologies to monitor HRV in older adults is still scarce, we believe those devices could be used for monitoring frailty in older adults. Long-term HRV monitoring is recommended to reduce artifacts produced by sensor disconnection or motion. On top of that, advanced signal processing such as nonlinear quantifications are considered more sensitive to aging-related problems such as frailty, and could therefore be used to minimize eventual erratic rhythms (41).

## Conclusion

Heart rate variability can be used as a potential marker for frailty because it helps to understand the changes in cardiac autonomic modulation. Using proper tools to monitor the heart rate variability would be ideal for the older adult population. In this sense, resources such as wearable devices are non-invasive and present advantages such as low cost and high benefit, representing an excellent tool to analyze the daily cardiac performance of the older adult population, thus making it possible to make a detailed monitoring.

## Author contributions

FA, VR, and RP conceived the design and concept. FA, VR, AL, JA, AF, DF, DM, IC, LO, and RP wrote the manuscript. All authors contributed to the editing and revision of the manuscript and approved the submission.

## Conflict of interest

The authors declare that the research was conducted in the absence of any commercial or financial relationships that could be construed as a potential conflict of interest.

## Publisher's note

All claims expressed in this article are solely those of the authors and do not necessarily represent those of their affiliated organizations, or those of the publisher, the editors and the reviewers. Any product that may be evaluated in this article, or claim that may be made by its manufacturer, is not guaranteed or endorsed by the publisher.
